# Does photoselective netting influence yield, chemical composition and antioxidant activities of essential oils in cultivated sage?

**DOI:** 10.3389/fpls.2025.1540520

**Published:** 2025-04-29

**Authors:** Lidija Milenković, Zoran S. Ilić, Ljiljana Stanojević, Ljubomir Šunić, Aleksandra Milenković, Jelena Stanojević, Dragan Cvetković

**Affiliations:** ^1^ Faculty of Agriculture, University of Priština in Kosovska Mitrovica, Lešak, Serbia; ^2^ Faculty of Technology, University of Niš, Leskovac, Serbia

**Keywords:** *Salvia officinalis* L., essential oil, GC/MS analysis, yield, composition, antioxidant activity

## Abstract

Yield, chemical profile and antioxidant activity of sage (*Salvia officinalis* L.) essential oils (SEOs) isolated from shaded (pearl, red and blue color nets) or non-shaded plants have been investigated. Analysis of the results can be seen a slightly higher amount of sage essential oil (SEO) from the shaded leaves samples, with minor exceptions. The highest yield of SEO was obtained from the samples cultivated under the blue photo-selective nets (1.97 mL/100 g p.m.). A total of 38 different components were identified in sage and divided into 7 groups. The main components of SEO were cis-thujone (32.9-35.2%), camphor (19.0-25.6%), *trans*-thujone (8.6-13.1%) and 1,8-cineole (9.4-11.0%).The strong antioxidant activity of all tested samples showed SEO from shaded sage leaves grown under the blue photoselective net for the all incubation times (20.00-37.28 mL/100 g p.m.).These researches confirmed that sage responded positively to blue light shading through increased production of secondary metabolic products such as EOs.

## 1 Introduction

Sage (*Salvia officinalis* L.) is one of the most abundant plant species from the Lamiaceae family that is used for various purposes; in traditional medicine, in the kitchen as a spice, as an additive in beverages, food technology, etc. Composition of phytochemicals in sage plants, is strongly influenced by genetics and species variations, environmental factor and cultivation techniques (Zhumaliyeva et al., 2023). Environmental factors, like temperature and light, play a significant role for plant growth, physiological processes and biosynthesis of phenol compounds in sage leaves (Moshari-Nasirkandi et al., 2024).

In order to create more favorable conditions for plant production due to global warming, new techniques of culinary plant protection and covering, such as shading, with different type of nets, canbe applied (Ilić et al., 2022a, b; Milenković et al., 2019, 2021). Color and density of the weave (shading index) nets modify the light and affect the intensity and spectral composition. In addition to providing physical protection (hail; strong winds, sandstorms, protection from aerial pests, birds, and insects, which can be transmitters of viral diseases), they are directed at optimizing the desirable physiological effect on plants (Ilić and Fallik, 2017). During creation, various chromatic additives are incorporated into photoselective nets that selectively disperse solar radiation (UV radiation, visible and long) and/or directly transform light into diffuse light. Manipulation of the spectral composition aims to directly influence the desired physiological response, while diffuse light enhances penetration light into the interior of the plant mass (Arthurs et al., 2013; Ilić et al., 2022a, b). Thus, black, grey and white nets reduce the light quantity (neutral shade), while red, blue, yellow and pearl nets change the red and blue light composition (photo-selective shade) (Oliveira et al., 2016). In addition, pearl, white, red, blue and yellow nets increase the scattered light ratio at luminous environment of cultivated plants (Ilić and Fallik, 2017; Shahak et al., 2004).

The benefit of colored nets as a means of light quality management includes changing leaf morphology, structure, improving quality and increasing yield and overall agro-economic performance and extending the harvest time of medicinal species (Kumar et al., 2013; Zhang et al., 2022). Different studies on the effect of shading (shade index and the color of shading nets) on the yield of essential oil suggest that each plant species reacts uniquely to light intensity. Light modification by nets has very important effect in culinary plant production and synthesis of secondary metabolite, such as essential oils. Shading plants by photo-selective shade nets synthesized more EOs than plants exposed to full sun light (Şeker et al., 2023). Light modification could improve the quantity and quality of essential oils in different medicinal plants (Ilić et al., 2022a; Milenković et al., 2021).

The lowest accumulation of essential oils in sweet basil was observed in the unshaded-control plants while the highest oil accumulation was achieved in plants from shade nets (Milenković et al., 2021). Marjoram and oregano tolerate shading well and gave higher essential oil yield when cultivated under shade. Lemon balm, mint, and sweet basil produce higher essential oil content under shaded conditions (Ilić et al., 2022a). Plants grown in the open field without shading exhibit lower essential oil content and reduced antioxidant activity (Ilić et al., 2022a). Average essential oil content from shade-exposed (40% shade index) sage (*Salvia officinalis* L.), oregano (*Origanum onites* L.) and rosemary (*Rosmarinus officinalis* L.) plants increased by 23% in first and 41% in second year of the experiment (Şeker et al., 2023).

Medicinal plants respond differently to individual color nets in terms of yield and EO chemical composition. Thus, *Melissa officinalis* L. achieved higher EO yield under blue nets (Oliveira et al., 2016), while basil obtained best results by red nets (Milenković et al., 2019). Similarly, thyme, marjoram, and oregano, achieved higher EO content with pearl nets (Milenković et al., 2021).

The content of essential oils is influenced by their origin, or the conditions in which the plant grew. Thus, cultivated *S. officinalis* had stronger antioxidant activity compared to wild sage EO (Ilić et al., 2023a). Phenolic content and antioxidant activity of the sage extracts depend on the extraction solvent and plant harvest season. Plants harvested in summer had better activity, while the ethanolic extract was the most active (Duletić-Laušević et al., 2016). EOs from sage as a natural food additive has the potential to reducing lipid oxidation and prolongs shelf life of various foods (Ben Akacha et al., 2024).

The aim of this research was to investigate the effects of pearl, blue, and red shade nets on leaf productivity of S*. officinalis* to better understand the effectiveness of this technique to enhance higher EO yield, composition and antioxidant activity.

## 2 Materials and methods

### 2.1 Plant material and experimental plan

The experiment with cultivated sage (from local domestic cultivar in Belgrade Seed-Seeds Company) was carried out in an experimental garden in the village of Moravac in south Serbia (21°42′ E, 43°30′ N, altitude 159 m a.s.l.) between 2020 and 2022.

The results of the agrochemical analysis of the alluvial soils type from **Table 1**. show that soil is highly accumulative with a fairly high humus content of 3.59% and very well supplied with phosphorus and potassium (40 mg/100g). After deep plowing in the autumn and pre-sowing soil preparation in the spring, two-month-old seedlings were transplanted at a distance of 70 cm between the rows and 40–50 cm in each row.

**Table 1:** Chemical analysis of soil (0-30 cm dept).

pH in 1M KCl	pH in H_2_O	CaCO_3_ %	Humus %	N %	P_2_O_5_	K_2_O
					mg/100g	
6.62	7.54	0.69	3.59	0.18	40	40

Spring planting is done early, as soon as the weather permits. Planting in the spring should not be delayed because, in the event of a drought, a large number of seedlings will die. In a permanent place, it is planted in rows at a distance of 50 to 60 cm and between plants in a row at 30-40 cm. An area of 1 ha requires 45,000-65,000 seedlings. Sage care consists in thinning and filling empty places, dusting, hoeing, fertilizing, protection against diseases and pests, etc. Special attention should be paid to plant care in the first year of cultivation, because then you need to help the plant to develop and spread as much as possible. In order to protect the plants from excessive temperatures and light during the summer months, the plants were covered horizontally 2m above the plants with colored (pearl, red and blue) shading nets (manufacturing from Polysack, Israel) with a shade index of 40%. They are built to selectively transmit the different spectral components of solar radiation (UV radiation, visible and long) and/or directly transform light into diffuse-scattered light. Light quality modification (light transmittance and scattering) by different shade nets is illustrated in **Table 2**.

**Table 2:** Light quality modification in the UV-B to far-red spectral range by color nets.

Net	Enriched Spectral Bands	Reduced Spectral Bands	Light Scattering
Blue	B	UV + R + FR	++
Red	R + FR	UV + B + G	++
Yellow	G+ Y + R + FR	UV + B	++
White	B + G + Y + R + FR	UV	++
Pearl		UV	+++
Grey	–	All to same extent	+
Black (Control)	–	All to same extent	–

Source: Rajapakse and Shahak, 2007.

Each of the colored shade nets specifically modifies the transmitted light spectrum in the ultraviolet, visible and far-red regions, enriching the relative content of scattered light and affects its thermal components (infrared region), in the function of the chromatic additives of plastic scattering elements and weaving design (Shahak et al., 2004). Effect of color nets on growing environment at typical day in July was presented at **Table 3**.

**Table 3:** Effect of different shade nets on plant environment (PAR, Solar radiation and temperature) on typical summer day in July.

Time (h)	PAR^*^ (μmol m^-^² s^-^¹)				Solar radiation W m^-2^			Blue	Temperature ^0^C	
	Non- shading	Pearl	Red	Blue	Non-shading	Pearl	Red		Non-shading	Shading Reduction
6:00	179.3	71.7	82.0	60.3	103.8	44.5	39.6	42.3	19.7	0.0
9:00	1403.0	843.6	854.4	673.3	513.8	294.6	311.3	301.4	29.7	+2.15
12:00	2090.8	1194.6	1268.3	1103.6	909.1	568.0	603.7	540.1	36.6	+0.8
15:00	1730.9	1063.3	1101.4	899.0	814.2	382	442.1	371.8	40.2	+0.17
18:00	679.3	379	399.7	314.4	391.5	109.1	118.4	105.7	38.8	+0.51

Combinations of shaded sage and non-shaded sage control plants were replicated three times in a split-plot design. Photosynthetically active radiation (PAR) above the canopy, using a Ceptometer Sun scan (SS1-UM-1.05; Delta-T Devices Ltd, Cambridge, UK). Readings are in units of PAR quantum flux (μmol m^−2^ s^−1^).

The Solarimeter – SL 100 (KIMO, Montpon, France) is an easy-to-use portable autonomous solarimeter that measures solar irradiation ranging from 1 Wm^−2^ to 1300 Wm^−2^.

During the three hottest summer months, when the shading nets are in place, solar radiation is significantly reduced by 35 to 45%, depending on seasonal variations and the color of the nets. The temperature beneath the shading nets is slightly lower (0.5–1°C) compared to the open field (**Table 4**). Under these conditions, sage plants grow more uniformly, benefiting from an environment that promotes greater biomass production and a higher content of essential oils.

**Table 4:** The impact of shading nets on solar radiation and temperature during the three summer months.

Nets	Month average solar radiation			Month average temperature °C		
	june	july	august	june	july	august
Pearl	434,2	502,4	434,2	28.05	30.2	28.95
Red	460,3	538,2	489,0	28.07	30.4	28.05
Blue	405,3	485,1	420,3	27.8	30.05	28.67
Control	713,8	843,6	756,1	28.5	30.8	29.90
*σ* standard deviation	123,022	96.51	135.9	0.251	0.271	0.458

Upon the appearance of the first flower buds, the plant height was measured. The thickness of the length of the leaf and weight leaf was measured over the first pair of leaves and the number of lateral branches – recorded.

Harvest must not be done while the crop is wet from dew or rain. It should be harvested in good and dry weather. Dry in a protected area in a draft, or in a dryer at a temperature of up to 40^0^C. Store the dried leaf in a cool and dark place. It is packed in paper or jute bags.

### 2.2 Essential oil extraction

Dried sage plants (100 g) and purified water (1000 mL) was combined in a flask attached to the condenser of Clevenger type hydro-distillation equipment, as described in the European Pharmacopoeia (Council of Europe, 2004).

### 2.3 GC/MS and GC/FID analysis

The details of the gas chromatography–mass spectrometry (GC/MS) and gas chromatography–flame ionization detection (GC/FID) analyses were given in our previous research by Milenković et al. (2021) and Ilić et al. (2022a). GC/MS analysis was performed on Agilent Technologies 7890B gas chromatograph, equipped with nonpolar, silica capillary column, HP-5MS (5% diphenyl- and 95% dimethyl-polysiloxane, 30 m × 0.25 mm, 0.25 μm film thickness; Agilent Technologies, Santa Clara, CA, USA) and coupled with inert, selective 5977A mass detector of the same company. The essential oils obtained were dissolved in diethyl ether. Solution prepared (1μl) was injected to the GC column through a split/splitless inlet set at 250^0^C in 40:1 split mode. Helium was used as the carrier gas, at a constant flow rate of 1 cm^3^/min. The oven temperature increased from 60^0^C to 246^0^C at the rate of 3^0^C/min. Temperatures of the MSD transfer line, ion source and quadrupole mass analyzer were set at 300^0^C, 230^0^C and 150^0^C, respectively. The ionization voltage was 70 eV and mass range *m*/*z* 41-415.

Data processing was performed using MSD ChemStation, MassHunter Qualitative Analysis and AMDIS_32 softwares (Agilent Technologies, USA). Retention indices of the components from the analyzed samples were experimentally determined using a homologous series of n-alkanes from C_8_-C_20_ as standards. Oil constituent’s identification was based on the comparison of their retention indices (RI^exp^) with those available in literature (Adams, 2007) (RI^lit^);

### 2.4 Antioxidant activity –DPPH assay

The antioxidant activity of the EO was determined using the DPPH assay. Essential oil was dissolved in the ethanol and a series of different concentrations was prepared. Ethanol solution of DPPH radical (1 cm^3^, 300 μmol solution (3x10^-4^ mol/L) was added to 2.5 ml of the prepared essential oil solutions. The details of the method used are provided by Stanojević et al. (2015). Essential oil concentration needed for the neutralization of 50% of the initial DPPH radical concentration is called EC_50_ value. This value was determined by using linear regression analysis of the different concentrations range of essential oil added to the reaction mixture.

### 2.5 Statistical analysis

Statistical analysis was performed using one-way ANOVA to assess the effect of shade net treatments. Statistical software STATISTICA 14 (TIBCO Software Inc. (2020). Data Science Workbench, version 14. http://tibco.com.) was used for all analyses. The LSD test was used to compare differences between means.

## 3 Results and discussion

### 3.1 Climatic conditions

Color shading nets, alongside physical protection (from birds, insects, hail, wind, various vectors, and disease carriers), reduce light intensity and modify its spectral composition to create specific microclimatic conditions that promote plant metabolism and the desired physiological processes influenced by light. The use of colored nets altered both the intensity and quality of light. While variations in light intensity have been somewhat documented (specifically in global radiation, though it is essential to focus on photo-biologically relevant radiation), the changes in light quality remain less well-defined.

During a sunny day in July in an open field without shading, the maximum solar radiation of 909 Wm^-2^ was recorded. It is evident; the reduction of net radiation due to the application of colored nets compared to the control. The greatest decrease in radiation intensity was recorded within the blue nets (540 Wm^-2^). Blue nets with 40% shading (1103μmol s^−1^m^2^) reduce the more intensively photosynthetically active radiation (PAR) compared to the other nets and control-open field condition (2090 µmol m^−2^ s^−1^).

Crop shading results in numerous changes in the microclimate but also in plant activity. These microclimate changes are related to CO_2_ exchange, assimilation, and thus indirectly to the growth and development of plants (Stagnari et al., 2018) and secondary metabolite biosynthesis (Ilić et al., 2023b).

#### 3.1.1 Plant morphology

Leaf morphology was significantly affected by shade treatment. Plants grown under red and blue shade nets had the largest leaves, while the leaves of plants grown under full sunlight were the smallest.

Shading had a significant effect on plant height (p < 0.05) with plants grown under red and blue being taller than pearl treatments and those grown under full sunlight being shortest. The number of main branches in red and blue shade nets was significantly greater than treatments with pearl nets and non shaded plants (p < 0.05). The influence of irradiance on fresh biomass was evident throughout this study. The highest fresh biomass was obtained in plants grown under red and blue color nets, with dry biomass exhibiting a similar response (**Table 5**). The mean values of fresh/dry ratio during the 3 month experiment are shown in **Table 5**. Ratio increased from blue follow red to pearl shade nets, but decreased in non shaded.

**Table 5:** Effect of color shade nets on sage morphology leaf and total yield.

Shade net	Height of plants (cm)	Number of main branches	Weight of leaf (cm)	Height of leaf (cm)	Fresh mass/plant (g)	Fresh yield kg/ha	Dry yield kg/ha	Fresh/ Dry ratio
Pearl	58.2a	13.9b	1.80a	8.80a	218.5a	12126.0a	3598.44a	3.37
Red	61.3a	14.3b	1.84a	9.33a	245.76a	13693.96a	4099.98a	3.34
Blue	55.4a	13.7b	1.70a	8.91a	237.46a	13179.03a	3951.58a	3.31
Control	42.3b	16.5a	1.73a	7.60b	167.03b	9389.0b	2876.3b	3.26

Values followed by different letters are significantly different at p < 0.05.

### 3.2 Essential oil yield

Differences in SEOs content were characterized by agro-ecological and grown condition, origin of eco-types or populations, plant age and harvest time, method of production (shading or non-shading condition), drying method and storage condition, method of extraction, etc.

Essential oil yield influenced by environmental factors; solar radiation is one of the most important parameter. The quantity and quality of light affect plant growth, development and sage essential oil yield and composition. The yield of sage essential oil (SEO) does not differ between plants from the open field - without shading (1.67mL/100) and those under red nets(1.67 mL/100) and pearl nets (1.65 mL/100). A statistically significantly higher SEO yield compared to other treatments was achieved in plants shaded with blue nets 1.98mL/100 g p.m. (**Table 6**).

**Table 6:** Effects of color shade nets on yield of sage essential oils (SEO).

Shade nets	Essential oil yield, mL/100 g plant material
Control (unshaded)	1.67±0.014a
Pearl nets	1.65±0.017a
Red nets	1.67±0.020a
Blue nets	1.98±0.025b*
Shade nets	*

*Values followed by different letters are significantly different at p < 0.05.

In our recent exploration the yield of EOs from wild sage plants (3.51 mL/100 g) was higher than that from cultivated plants(shaded plants: 3.20 mL/100 g and non-shaded plants: 2.56 mL/100 g),(Ilić et al., 2023a). Similarly, *Melissa officinalis* plants grown under shade from blue net showed the highest essential oil yield and red net resulted in a reduction in the yield of essential oil in leaves (Oliveira et al., 2016).

The yield of EOs of thyme, marjoram, and oregano obtained from non-shading plants was 2.32, 1.51, and 0.27 mL/100 g of plant material, respectively. At the same time under shading conditions (40% shaded index) plants synthesized more EOs (2.57, 1.68, and 0.32 mL/100 g of plant material) (Milenković et al., 2021). Different medicinal species react specifically to the light modification by nets. Thyme and lemon balm covered by nets produced higher levels of EOs but shading degree content of EO in mint plants (Ilić et al., 2021). The use of colored shade nets during the growth of different medicinal plants provides spectral changes, resulting in a higher content of EOs in sweet basil (Milenković et al., 2021; Ilić et al., 2021) mint, oregano, marjoram, thyme (Ilić et al., 2022a; Milenković et al., 2021) lemon balm (Ilić et al., 2022a; Oliveira et al., 2016) and sage (Ilić et al., 2023b).

Level of shade intensity also effect SEO yield. The yield of essential oil was highest in sage plants grown under 30% shade and decreased in full sunlight and with increasing shade levels (70%) (Rezai et al., 2018). Essential oil rate increased in sage, oregano and rosemary under low-light conditions (40% shade index) (Şeker et al., 2023).

The biosynthesis of aromatic compounds occurs through two complex chemical pathways, involving different enzymatic reactions which depend on a large group of enzymes known as terpene synthases (Rehman et al., 2015). Light spectrum variations can be used for the biosynthesis of substances in plants including essential oils, through the stimulation of photosensitive enzymes involved in the mevalonic acid pathway. Thus, irradiance can directly influence the production of essential oils, or indirectly, through the increase of plant biomass (Pegoraro et al., 2010).

The comparatively low biomass produced by plants cultivated under full sunlight, and consequently low essential oil content, suggests that assimilates might have been directed to the maintenance and repair mechanisms necessary to support high irradiance conditions.

### 3.3 Essential oil composition

#### 3.3.1 Essential oil composition of sage

**Figure 1:**
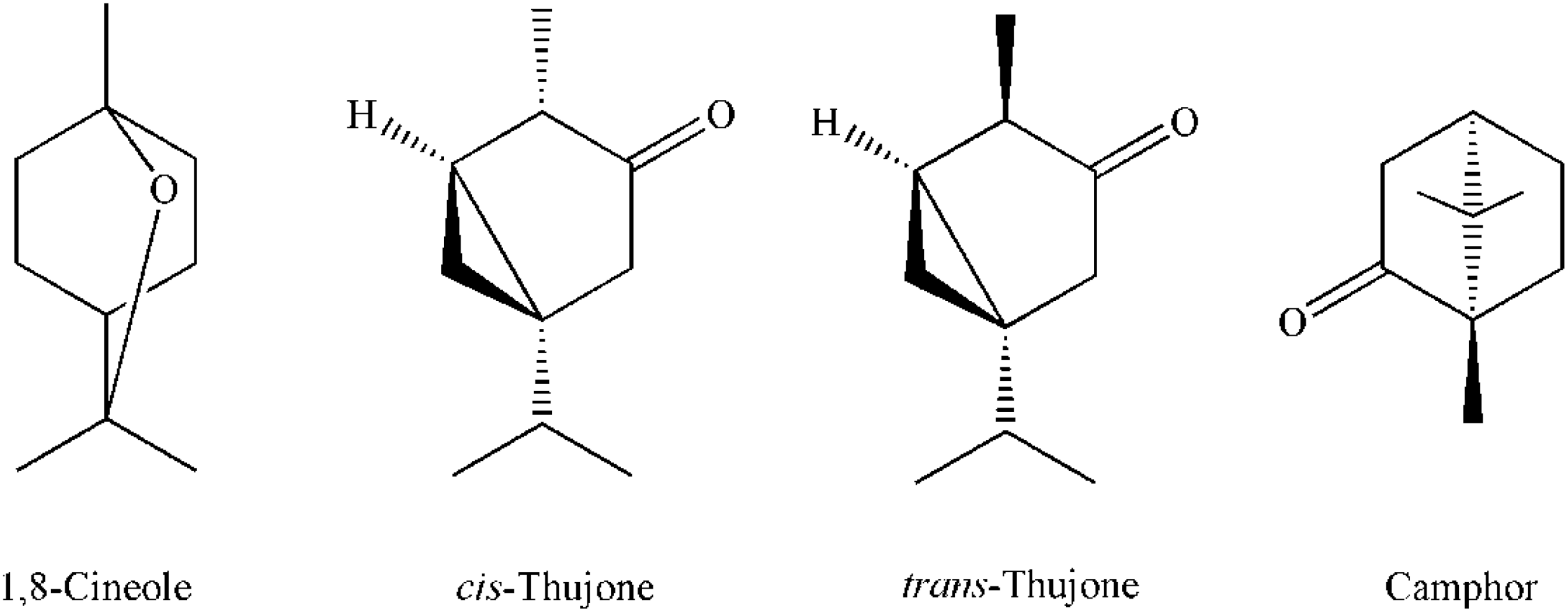
Chemical structures of the most abundant components in sage essential oil.

Duration and intensity of light affects the accumulation of some compounds and has significant effects on plant photochemistry. In our study *cis*-thujone (32,9-35.2%), camphor (19.0-25.6%), *trans*-thujone (8.6-13.1%), 1-8cineole (9.4-11.0%), camphene (3.8-5.8%) and α-pinene (3.5-4.3%)where the most commonly components in sage essential oil (SEO) as shown in **Table 7**. The chemical structures of the most abundant components in sage essential oil are presented in **Figure 1**.

**Table 7:** Influence of color shade nets on chemical composition of sage essential oil (SEO).

N°	*t*_ret_, min	Compound	RI^exp^	RI^lit^	Method of identification	Content %			
						Non shade Control	Color shade nets		
							pearl	red	blue
1.	6.31	Tricyclene	920	921	RI, MS	0.2 ± 0.000	0.1 ± 0.000	0.2 ± 0.001	tr
2.	6.41	α-Thujene	923	924	RI, MS	0.2 ± 0.000	0.2 ± 0.001	0.2 ± 0.000	0.2 ± 0.001
3.	6.63	α-Pinene	931	932	RI, MS	4.3 ± 0.016	3.5 ± 0.010	3.5 ± 0.008	3.9 ± 0.003
4.	7.08	Camphene	946	946	RI, MS	5.2 ± 0.018	4.8 ± 0.016	5.8 ± 0.020	3.8 ± 0.020
5.	7.79	Sabinene	971	969	RI, MS	0.1 ± 0.000	0.1 ± 0.000	tr	tr
6.	7.92	β-Pinene	975	974	RI, MS, Co-I	2.1 ± 0.007	1.8 ± 0.008	2.1 ± 0.008	1.8 ± 0.005
7.	8.09	1-Octen-3-ol	981	974	RI, MS	0.2 ± 0.000	tr	tr	tr
8.	8.32	Myrcene	989	988	RI, MS	1.1 ± 0.002	1.0 ± 0.001	1.2 ± 0.002	1.1 ± 0.001
9.	8.82	α-Phellandrene	1005	1002	RI, MS	tr	tr	tr	tr
10.	9.22	α-Terpinene	1016	1014	RI, MS	0.2 ± 0.000	0.2 ± 0.005	0.2 ± 0.000	0.2 ± 0.001
11.	9.55	*p*-Cymene	1024	1020	RI, MS	0.3 ± 0.003	0.3 ± 0.003	0.3 ± 0.001	0.3 ± 0.001
12.	9.67	Limonene	1028	1024	RI, MS, Co-I	tr	tr	tr	tr
13.	9.76	1,8-Cineole	1028	1026	RI, MS, Co-I	10.4 ± 0.060	9.4 ± 0.032	11.0 ± 0.035	10.8 ± 0.042
14.	10.76	γ-Terpinene	1057	1054	RI, MS	0.4 ± 0.004	0.4 ± 0.004	0.4 ± 0.002	0.4 ± 0.0000
15.	11.26	*cis*-Sabinene hydrate	1070	1065	RI, MS	0.2 ± 0.000	tr	tr	tr
16.	11.38	*cis*-Linalool oxide (furanoid)	1073	1067	RI, MS	tr	tr	tr	tr
17.	11.90	Terpinolene	1087	1086	RI, MS	0.2 ± 0.000	0.2 ± 0.005	0.2 ± 0.000	0.2
18.	12.07	*p*-Cymenene	1092	1089	RI, MS	–	–	–	tr
19.	12.79	*cis*-Thujone	1110	1101	RI, MS	32.9 ± 0.043	33.0 ± 0.042	35.2 ± 0.051	34.4 ± 0.030
20.	13.18	*trans*-Thujone	1120	1112	RI, MS	12.2 ± 0.045	11.6 ± 0.032	8.6 ± 0.034	13.1 ± 0.030
21.	14.15	iso-3-Thujanol	1143	1134	RI, MS	–	tr	tr	tr
22.	14.34	Camphor	1146	1141	RI, MS, Co-I	19.4 ± 0.064	25.6 ± 0.080	23.5 ± 0.064	19.0 ± 0.030
23.	14.91	*trans*-Pinocamphone	1161	1158	RI, MS	0.2 ± 0.000	tr	tr	tr
24.	15.36	Borneol	1172	1165	RI, MS, Co-I	1.7 ± 0.006	2.0 ± 0.004	1.9 ± 0.004	1.8 ± 0.004
25.	15.74	Terpinen-4-ol	1181	1174	RI, MS	0.5 ± 0.005	0.4 ± 0.004	0.4 ± 0.003	0.5 ± 0.005
26.	16.43	α-Terpineol	1196	1186	RI, MS	0.2 ± 0.000	tr	tr	tr
27.	16.60	Myrtenol	1202	1194	RI, MS	0.2 ± 0.000	tr	tr	tr
28.	20.09	Isobornyl acetate	1283	1283	RI, MS	0.4 ± 0.004	0.4 ± 0.000	0.5 ± 0.006	0.4 ± 0.001
29.	20.42	*trans*-Sabinyl acetate	1291	1289	RI, MS	0.2 ± 0.000	tr	tr	tr
30.	25.26	Methyl eugenol	1407	1403	RI, MS	tr	–	–	–
31.	25.71	(*E*)-Caryophyllene	1417	1417	RI, MS	0.7 ± 0.004	0.8	0.8	0.9
32.	27.10	α-Humulene	1452	1452	RI, MS	1.1 ± 0.01	1.2 ± 0.008	1.2 ± 0.006	1.8 ± 0.008
33.	29.99	Myristicin	1526	1517	RI, MS	1.5 ± 0.004	–	–	–
34.	31.22	Elemicin	1557	1555	RI, MS	0.3 ± 0.003	–	–	–
35.	32.22	Caryophyllene oxide	1583	1582	RI, MS	0.3 ± 0.003	tr	tr	0.3 ± 0.001
36.	32.65	Viridiflorol	1595	1592	RI, MS	1.7 ± 0.016	1.7 ± 0.006	1.6 ± 0.016	2.9 ± 0.008
37.	33.22	Humulene epoxide II	1610	1608	RI, MS	0.5 ± 0.006	0.4 ± 0.001	0.4 ± 0.001	0.6 ± 0.006
38.	48.21	Manool	2052	2056	RI, MS	1.0 ± 0.001	0.9 ± 0.009	0.7 ± 0.007	1.4 ± 0.004
					Total identified(%)	100.0	100.0	100.0	100.0	
Grouped components (%)									
Monoterpene hydrocarbons (1-6, 8-12, 14, 17)						14.3 ± 0.050	12.6 ± 0.053	14.3 ± 0.042	12.0 ± 0.018
Oxygen-containing monoterpenes (13, 15, 16, 18-27)						78.4 ± 0.61	82.5 ± 0.199	81.0 ± 0.191	80.00.143
Sesquiterpene hydrocarbons (29, 30)						1.8 ± 0.004	2.0 ± 0.000	2.0 ± 0.006	2.8 ± 0.001
Oxygen-containing sesquiterpenes (33-35)						2.5 ± 0.006	2.1 ± 0.0010	1.9 ± 0.008	3.8 ± 0.016
Phenylpropanoids (28, 31, 32)						1.8 ± 0.000	–	–	–
Diterpenoids (36)						1.0 ± 0.003	0.9 ± 0.000	0.7 ± 0.000	1.4 ± 0.000
Others (7)						0.2 ± 0.007	tr	tr	tr

*t*_ret._: Retention time; RI^lit^-Retention indices from literature (Adams, 2007); RI^exp^: Experimentally determined retention indices using a homologous series of *n*-alkanes (C_8_-C_20_) on the HP-5MS column. MS, constituent identified by mass-spectra comparison; RI, constituent identified by retention index matching; Co-I, constituent identity confirmed by GC co-injection of an authentic sample; tr, trace amount (<0.05%).

Thirty six compounds of EO were identified in nonshading - open field sage, mainly oxygen-containing monoterpenes (78.4%), monoterpene hydrocarbons (14.3%), oxygenated sesquiterpenes(2.5%), sesquiterpene hydrocarbonsand phenylpropanoids (1.8%), diterpenoids (1.0%) and others (0.2%). SEO from plant grew under different photoselective shade nets contained thirty-four components.

Color shade nets have a distinct effect on the composition of various components in sage essential oils (SEO). The highest concentration of cis-thujone was observed in plants covered with red nets (35.2%), while the highest level of trans-thujone was found in plants under blue nets (13.1%). Camphor was most abundant in plants covered with pearl nets (25.6%). Quality and quantity of light affects phytochemical accumulation metabolism. Light signals are perceived by photoreceptors and they regulate the accumulation of various phytochemicals depending on some stress factor and light (Oh et al., 2014). The effect of light composition on terpene biosynthesis has been reported in several plants, including aromatic herbs such as peppermint and basil, which are rich in essential oils. Previous studies have investigated changes in terpene composition or emissions (in plants lacking specific storage structures) under light treatments in the UV, blue, red, and far-red regions of the light spectrum. Many studies manipulating the red and blue spectral composition, by the use of the color shade nets, have been shown to influence the production of monoterpenes and sesquiterpenes (e.g., Pennisi et al., 2019; Morello et al., 2022). Additionally, there is evidence of a negative correlation between blue light intensity and monoterpene production (Pallozzi et al., 2013), similar to this study. Also, the role of blue light in terpene biosynthesis has also been highlighted in several studies (e.g., Maffei and Scannerini, 1999; Amaki et al., 2011).

In our previous study, significant differences were observed in the main constituents of the essential oil (EO) between wild and cultivated sage. The primary components of the EO are cis-thujone, camphor, and 1,8-cineole. The content of the toxic cis-thujone in sage (23.5%) was reduced in shaded plants compared to those grown in open fields (cis-thujone 28.3%) (Ilić et al., 2023a).

Similarly research by Radulović et al. (2017)with natural sage populations in Serbia revealed that α-thujone (28.2%) was the main EO component, while β-thujone occupied only 5.1%.In research Ilić et al. (2023a) have found that sage can be classified into two groups based on thujone content; (1)wild Montenegrin sage with high (48.8%) total thujone content and (2) cultivated sage from Serbia with medium thujone content (32.6%) resulting in different rations between *cis*and *trans*-thujones (*cis/trans* 8:1 in wild and 6:1 to 2.5:1 in cultivated sage) (Ilić et al., 2023a).

α-thujone content in SEO was in negative linear relation with shading level while in contrast, carvacrol content of oregano essential oil increased by shade treatment. The results concluded that quality properties of SEO from shaded plants were promising, considering their potential in intercropping systems (Ilić et al., 2022b).

### 3.4 Antioxidant activity - DPPH test

The oxygen-containing monoterpenes and monoterpene hydrocarbone are the most responsible for antioxidant activity. The most prominent constituents, cis-thujone and camphor, contribute to the increased antioxidant activity of the sage essential oil (SEO).

The lowest antioxidant activity (AA) of EO in sage plants under pearl nets is significantly lower compared to other nets. The antioxidant activity of EO in plants shaded with red nets and plants without shading from the open field is at the same level and statistically not significant (**Table 8**).

**Table 8:** Influence of shading sage by color nets and time of incubation on EO antioxidant activity.

	Essential oil EC_50_, mg/mL		
	20 min incubation	40 min incubation	60 min incubation
Control (non shaded)	46.61 ± 0.183b	31.03 ± 0.108b	24.28 ± 0.073b
Pearl net	56.16 ± 0.220c	38.63 ± 0.168c	31.14 ± 0.096c
Red net	46.82 ± 0.404b	31.54 ± 0.138b	24.30 ± 0.081b
Blue net	37.28 ± 0.251a	26.16 ± 0.098a	20.00 ± 0.115a
Shade nets	***	***	***

Means in columns marked with same letter are not statistically different; ***—differences are significant.

The strongest antioxidant activity of EO was recorded in the sage plants that grew under blue photoselective nets for the all incubation times (20.00-37.28 mL/100 g p.m.). This research confirmed that under shading conditions with blue nets, sage redirects its metabolism towards increased essential oil production and enhanced antioxidant activity. The antioxidant activity of SEO improves as the incubation time is extended from 20 to 40 and 60 minutes (**Table 8**).

Cultivated sage was found to have stronger antioxidant activity (shaded plants 6.16 mg/mL or non-shaded 7.49 ± 0.13 mg/mL) compared to wild sage plants (Ilić et al., 2023a). Antioxidant activity of SEO has already been found in studies from other countries. Thus, SEO of the plants from Tunisia showed an EC_50_value of 6.7 mg/mL (Ben Khedher et al., 2017). An EC_50_value of 28.28 ± 10.241 was reported for extracts of sage from Serbia and an IC_50_ 48.62 ± 29.181 from Montenegro (Duletić-Laušević et al., 2016). Lalević et al. (2023) in similarly study with shading of different medicinal plants present that the EC_50_ values (efficient concentration of the oil, the smaller the EC_50_ value - the better the antioxidant activity) increased in shading condition only in lemon balm plants. No increased antioxidant activity observed in thyme and mint shaded plants. Collection, diversification and preservation of the most resistant populations and ecotypes of *Salvia officinalis* with high level of EO before they disappear and lost during the global warming, are very urgent. Also, it is necessary to combine new cultivation methods that optimize the concentration of particular volatile compounds (Ilić et al., 2023b). Light and temperature with plant density create special microenvironment during the growth and development of sage plants (due to moderate radiation and plant photosynthesis) have a strong influence on the relationship between biomass production and essential oil content and composition. Based on previous results where artificial shading conditions for plant growth were created using shading nets, research under natural shading conditions, involving the establishment of two or more crops with different growth habits combined in a consociation, should be conducted with sage in order to develop recommendations for this type of cultivation. Finally, our data showed that blue net could be incorporated into the protected cultivation practices currently used for producing *S. officinalis* essential oil. The application of EO in practice is multipurpose such as an antioxidant in pharmaceutical and cosmetic industries or antimicrobial agent in the food and processing industry.

## 4 Conclusion

Sage plants covered by blue shade nets produce the significantly highest essential oils yield (1.98 mL/100 g) in comparison with other nets and non-shading plants. Monoterpene hydrocarbons (*cis*-thujone, camphor, *trans*-thujone, 1-8cineole) was predominant constituents in the sample grown under photoselective nets, while the highest content of monoterpenehydrocarbons (α-pinene and camphene) recorded in plants from open field. The highest level of sesquiterpene hydrocarbons and oxygen-containing sesquiterpenes was obtained in the shading plants with blue nets. The modification of the light intensity via shade nets improves antioxidant activity (AA) in sage plants. The strongest AA recorded with blue nets (20.00 mL/100 g p.m.) after 60 min of incubation. Optimized production techniques using plant shading from the present study could provide useful methods for improving the content and composition of sage essential oils.

## Data Availability

The raw data supporting the conclusions of this article will be made available by the authors, without undue reservation.
